# Dissecting the Molecular Mechanism of Ionizing Radiation-Induced Tissue Damage in the Feather Follicle

**DOI:** 10.1371/journal.pone.0089234

**Published:** 2014-02-20

**Authors:** Xi Chen, Chunyan Liao, Qiqi Chu, Guixuan Zhou, Xiang Lin, Xiaobo Li, Haijie Lu, Benhua Xu, Zhicao Yue

**Affiliations:** 1 Institute of Life Sciences, Fuzhou University, Fuzhou, Fujian, China; 2 Department of Radiation Oncology, Union Hospital of Fujian Medical University, Fuzhou, Fujian, China; Center for Cancer Research, National Cancer Institute, United States of America

## Abstract

Ionizing radiation (IR) is a common therapeutic agent in cancer therapy. It damages normal tissue and causes side effects including dermatitis and mucositis. Here we use the feather follicle as a model to investigate the mechanism of IR-induced tissue damage, because any perturbation of feather growth will be clearly recorded in its regular yet complex morphology. We find that IR induces defects in feather formation in a dose-dependent manner. No abnormality was observed at 5 Gy. A transient, reversible perturbation of feather growth was induced at 10 Gy, leading to defects in the feather structure. This perturbation became irreversible at 20 Gy. Molecular and cellular analysis revealed P53 activation, DNA damage and repair, cell cycle arrest and apoptosis in the pathobiology. IR also induces patterning defects in feather formation, with disrupted branching morphogenesis. This perturbation is mediated by cytokine production and Stat1 activation, as manipulation of cytokine levels or ectopic Stat1 over-expression also led to irregular feather branching. Furthermore, AG-490, a chemical inhibitor of Stat1 signaling, can partially rescue IR-induced tissue damage. Our results suggest that the feather follicle could serve as a useful model to address the in vivo impact of the many mechanisms of IR-induced tissue damage.

## Introduction

IR is an important tool in cancer therapy, either as the primary choice or in combination with other chemotherapeutic agents [1]–[3]. The mechanism of IR-induced tissue damage is likely to be complex. At the cell level, virtually every organelle is affected [4], [5]. DNA double-strand break is a well-documented event, leading to a choice of cell survival or death [6]. Other events include the mitochondrial or ER responses, which may lead to cell stress and ROS production [7], [8]. At the tissue or whole-body level, the response is not limited to cell-autonomous. By-stand effect, or cell non-autonomous response, plays important roles [9], [10]. IR induces the expression of many cytokines, which may have a local or systematic impact [11]–[14]. Inflammation, and subsequent tissue fibrosis is manifested by these cytokines which may sustain for a long period of time. Finally, IR-induced tissue damage is often associated with active cell proliferation. Perturbation of the stem cell niche or depletion of the stem cells/progenitors has been reported [15]–[17].

Given the wide range of cell activities that might be disrupted by radiation exposure, it is important to evaluate their relative contributions in vivo. Careful choice of dose-regimen is critical in cancer therapy [18]–[20], because IR-induced tissue response is dose-dependent. For instance, would an IR-induced P 53 activation/DNA damage and repair response consistently lead to tissue damage? How would a cytokine response contribute to IR-induced early damage? At the cell level, apoptosis was believed to be the main cause of IR-induced tissue damage [5], [21], [22]. However, IR also induces significant cell cycle arrest [5], [23], [24], the contribution of which remains under-explored. Furthermore, the biological systems usually have tremendous repair capability as a defense mechanism. Which aspects of the defense are most vulnerable? To answer these questions, detailed analysis using in vivo models is critical.

Chicken embryo has a long history of serving as a model to dissect the mechanism of IR-induced tissue damage. IR has a profound impact on limb bud development, leading to truncation of the structure [25], [26]. Analysis of this phenomenon leads to an important notion regarding proximal-distal limb patterning, the progress zone model [27]. Recent study suggested that it was the specific ablation of cartilage progenitors that leads to limb bud truncation [28]. IR also has an impact on embryonic feather bud formation. A dose-dependent ablation of cells leads to reduced cell number, which then results in perturbed hexagonal patterning of the feather bud [29]. Recent discovery in the Chernobyl area, where radiation contamination remains to be an environment hazard, showed that the birds were affected with pigmentation abnormality and cancer development [30]. These results suggest that the birds are sensitive to radiation exposure and could serve as a model system to investigate the impact of radiation exposure.

The avian feather, like mammal hair, is an ectoderm organ with robust growth and regeneration capability but with much more complex structures [31]–[33]. These structures are characterized by regular epithelial branching (barbs) inserted onto a central shaft (rachis). The formation of the feather structure is regulated by a complex network of evolutionarily conserved signaling pathways. Any perturbation of feather development will be recorded in the final morphology. Physiologically, when an acute starvation occurred or food with pigment properties was supplied, an isochronic fault line, or an “isochrone” would form [32], [34]. Experimentally, when a specific signaling molecule was manipulated, the feather structure would also change accordingly [35]–[37].

Here we use the feather follicle as a model to dissect the impact of the many possible mechanisms of IR-induced tissue damage. We found a dose-dependent response on the feather morphology. Molecular and cellular analysis revealed DNA damage response and P 53 activation, cell cycle arrest and apoptosis in the feather follicle. Interestingly, we also found patterning defects in feather branching formation. This is due to elevated cytokine production and Stat1 activation. A chemical inhibitor of Stat1 signal transduction can reduce the defects and partially rescue the feather phenotypes. Therefore, an IR-induced cytokine response is a major cause of tissue damage in feather formation.

## Results

### IR Induces Defects in Feather Formation in a Dose-dependent Manner

**Figure 1:**
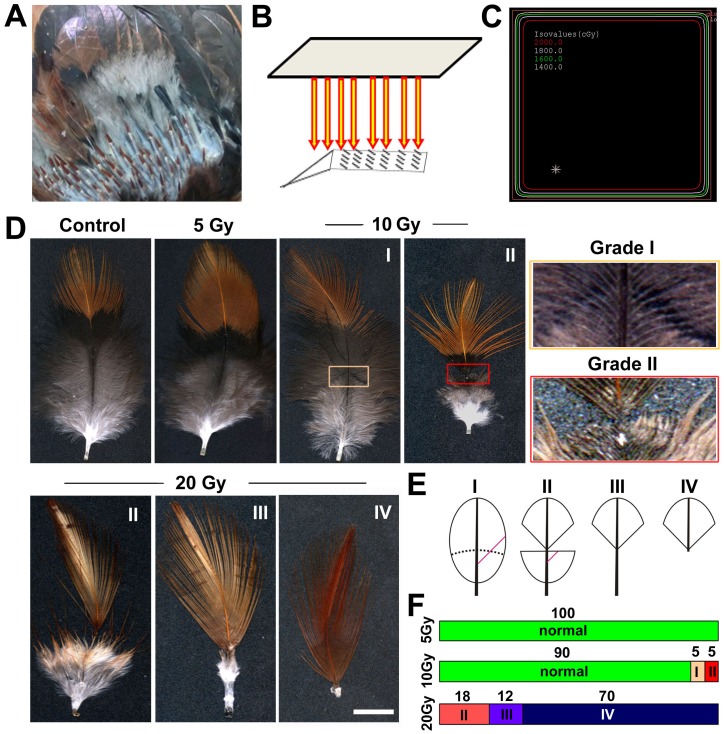
IR induces defects in feather formation in a dose-dependent manner. (A) Feathers in the chicken wing region were plucked 2 weeks earlier to induce active growth. (B) This area was placed under a lead window and exposed to IR. The rest of the body was protected. (C) Homogeneous dose distribution in this exposure window. (D) Phenotypes of feathers after IR exposure. Feathers were collected 3 weeks after exposure, and representative examples are shown. Higher magnifications showing the typical defects in feather morphology. (E) Schematics showing the different grade of defects in the feather. A red line indicates a barb that is retained in grade I defect, but is broken in grade II defect, and not formed in grade III/IV defects. (F) Statistics of the defects at different doses. Feathers from three birds were collected and analyzed for each experimental dose. n = 40 at 5 Gy, 40 at 10 Gy and 60 at 20 Gy. Bar = 1 cm.

We induced active feather growth in the wing contour region by plucking and regeneration. After 2 weeks the feathers entered active growth phase (Fig. 1A). The average growth rate could be 1–3 mm per day. We exposed this area to IR under a Varian Clinac 23ex machine that was used in clinic settings. The rest of the body was protected by a lead cover (Fig. 1B). A homogenous distribution of radiation dose was achieved in this window, as exampled by the 20 Gy treatment protocol (Fig. 1C). After treatment, the chickens were housed with care and feathers were collected 3 weeks later after finishing the growth cycle. Sometimes feathers in the next few cycles were also collected. Even at the highest dose used here (20 Gy), no systematic abnormality was noticed, and the chickens appeared normal during the whole experimental period up to 6–9 months.

We found IR perturbed feather formation in a dose-dependent manner. At 5 Gy, no abnormality was found in the feathers (Fig. 1D). After 10 Gy treatment, 10% feathers showed obvious defects (4/40). Among these, grade I defect was a typical “isochrone”, with continuous barbs remained. Grade II defect was characterized by broken barbs. Since the barbs need attach to the rachis otherwise they will be lost, a “V” shape was created. The feather growth resumed afterwards, suggesting a reversible impact. More severe defects were observed after 20 Gy treatment. In grade III defect, the upper edge remained a “V” shape, but feather growth was not recovered to normal level (6/50; 12%). In grade IV defect, the feather growth was terminated (35/50; 70%). A summary diagram shows the different types of defects (Fig. 1E), and a statistics of the dose-response is shown (Fig. 1F).

We also examined the recovery of these feather follicles in the next round of growth cycle. All feathers resumed growth and appeared normal. Most (80%) feathers showed a loss of pigmentation (Fig. S1), similar to those observed in the Chernobyl birds [30]. Therefore, stem cells of the epithelial/mesenchymal origin remained unperturbed or recovered, but melanocyte stem cells [38], [39] were depleted by 20 Gy treatment. All feathers appeared normal in the next cycle after 5 or 10 Gy treatment (not shown).

### Patterning Defects Induced by IR

**Figure 2:**
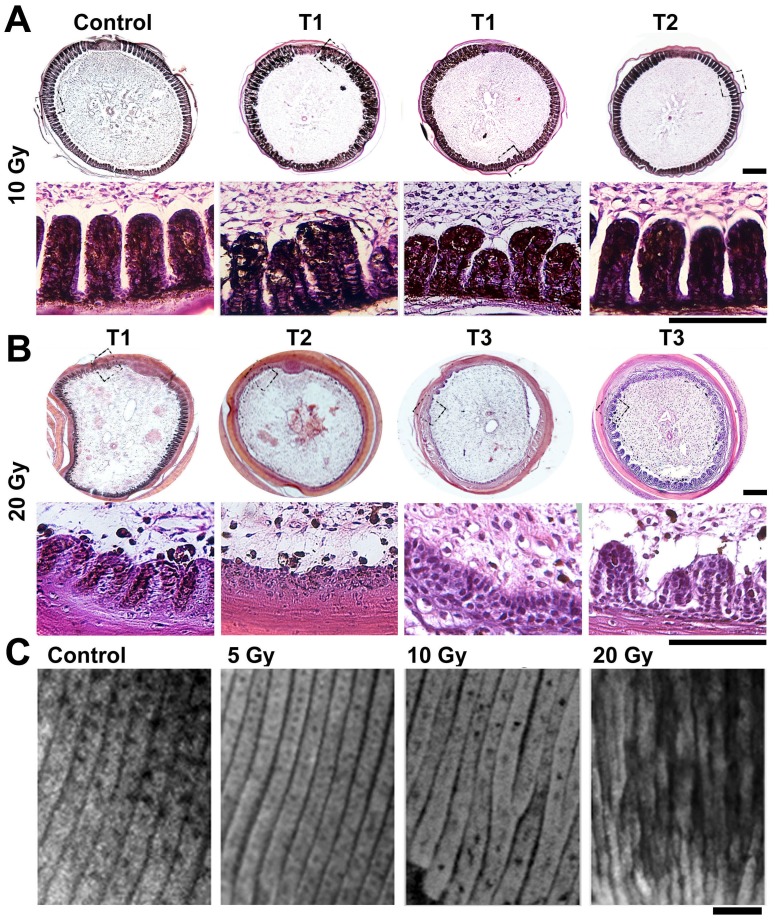
Analysis of IR-induced defects in feather formation. (A–B) H&E analysis of feather follicles after IR-exposure at different doses. At 10 Gy, the total epithelial cell number remains unchanged, yet extensive branching abnormality is seen. This is transient and reversible; at T2 (2 days post-IR) the feathers become normal again. At 20 Gy, the feather epithelium is significantly reduced. Heterogeneity in T3 (3 days post-IR) is noticed, with some recovered little while others showed branching epithelium again. Representative examples of 8 follicles examined in each case are shown. (C) Whole-mount prep of feather epithelial branching at T1 (1 day post-IR) showing the disrupted patterning at 10 or 20 Gy. Bar = 100 µm.

Histological analysis revealed more interesting insights. At 5 Gy, feather branches remained normal (Fig. S2). At 10 Gy, the total epithelial cell number was unchanged but the partitioning into each barb was perturbed (Fig. 2A). Among the 8 follicles analyzed, 87.5% (7/8) showed patterning defects in H&E analysis at day 1 post-treatment (T1). No major defects were found at day 2 post-treatment (T2). This high incidence, as compared with only 10% feathers showed morphological defects, suggests that not all patterning defects will result in a gross morphological consequence. The effect is transient and reversible, and possibly re-adjustable during later feather growth. On the other hand, IR-induced defects are actually more consistent and severe than analyzed by gross morphological observation.

At a higher dose (20 Gy), the impact was more severe (Fig. 2B). Epithelial cell number was significantly reduced, as compared to control or after 10 Gy treatment. Epithelial branching was shortened at T1, or not visible at T2. This reduction in feather epithelium was quite consistent: among the 8 follicles examined, all showed similar phenotypes. Heterogeneity in response was noticed at T3. In some follicles the epithelial recovery was not significant yet, while in others branching already resumed. Therefore, heterogeneity in response is mainly due to a difference in the recovery phase.

We examined the perturbed feather development by whole-mount prep analysis [36]. In this protocol, the feather follicle is cut open to expose the interior epithelial branching pattern. We found regular epithelial branching in control and 5 Gy samples, which became irregular at 10 Gy, and seriously disrupted in size and organization at 20 Gy (Fig. 2C).

### IR Activates P53, Induces Cell Death and Cell Cycle Arrest in the Feather Follicle

**Figure 3:**
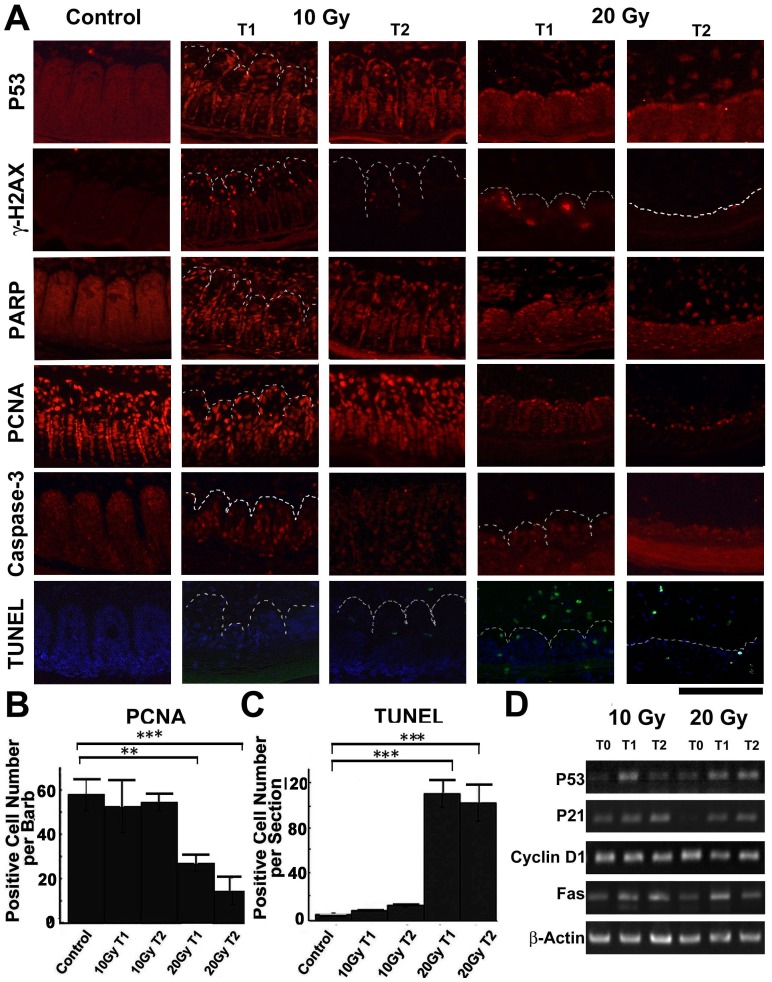
Molecular analysis in the feather follicles after IR exposure. (A) Immunohistochemistry (red) showing P53 activation, gama-H2AX expression, and PARP activation after IR exposure. Cell proliferation is indicated by PCNA staining, and cell apoptosis is monitored by Caspase-3 staining and TUNEL analysis (green). (B–C) Quantification of PCNA staining and TUNEL analysis. Significant decrease in PCNA staining is noticed after 20 Gy treatment, when TUNEL staining is also the most significant. **, p<0.01; ***, p<0.001. (D) RT-PCR analysis of gene expression in the feather follicle. T0, control samples before IR; T1, 1 day post-IR; T2, 2 days post-IR. Bar = 100 µm.

We next analyzed the molecular events after IR exposure in the feather follicle (Fig. 3A). A hallmark for IR-induced tissue response is P53 activation. This response is characterized by increased overall expression and condensed nuclear localization of P53 protein. Indeed we found P53 activation after 5 Gy treatment (Fig. S3), which became more obvious at 10 Gy and 20 Gy. IR also induces DNA damage and repair, which is characterized by gama-H2AX staining. As expected, we found distinct nuclear staining for this antibody at 5 Gy and upwards. RT-PCR analysis also revealed increased expression of *p53* and *p21* genes, suggesting activation of P53 signaling (Fig. 3D; quantified in Fig. S4). These results suggest that the feather follicle shows the typical responses to IR.

IR induces a wide range of possible cell responses, including DNA damage/repair, cell cycle arrest, and cell death/apoptosis. We analyzed these possibilities in the feather follicle. PARP is involved in DNA damage repair or as a substrate for Caspase-3 in apoptosis [40]. We found extensive nuclear localization of PARP after 5 Gy, 10 Gy or 20 Gy treatment. These results suggest that PARP is activated in response to IR, but is not necessarily related to cell apoptosis (see below). PCNA is an indicator of cell proliferation. IR-induced cell cycle arrest is shown by reduced PCNA staining. We found reduced expression of PCNA after 20 Gy treatment, but not at 10 Gy (Fig. 3B). This is consistent with the observation that 10 Gy treatment did not result in reduced epithelial cell number. On the other hand, Caspase-3 activation could indicate cell apoptosis. We found Caspase-3 was normally enriched in the tip of each barb, and in between barbs (the marginal plate), which is consistent with previous report [41]. IR activates Caspase-3, as indicated by increased nuclear localization. However, this activation does not necessarily lead to cell apoptosis, since 10 Gy treatment did not reduce the cell number. This is further confirmed by TUNEL analysis. We found very few positive TUNEL signal after 5 Gy or 10 Gy treatment. TUNEL staining became more significant at 20 Gy (Fig. 3C). More changes in molecular expression were analyzed by RT-PCR, including elevated *Fas*, but constant *Cyclin D1* gene expression (Fig. 3D and Fig. S4). These results suggest that cell cycle arrest and apoptosis become obvious only at a higher IR dose (20 Gy). At a lower dose (10 Gy), although P53/gama-H2AX/PARP/Caspase-3 is activated, the total cell number is not reduced.

### IR Activates Cytokine Production Which Disrupts Feather Patterning

**Figure 4:**
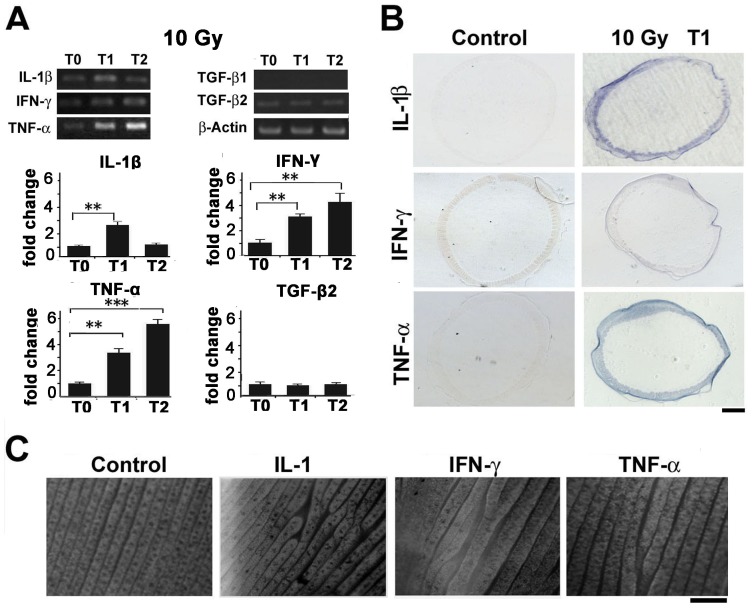
Cytokine gene expression and manipulation in the feather follicles. (A) RT-PCR analysis showing increased expression of IL-1beta, IFN-gama, TNF-alpha, but not TGF-beta1 or TGF-beta2 in the feather follicles. Densitometry of the results were quantified and statistically analyzed. **, p<0.01; ***, p<0.001. (B) In situ hybridization showing cytokine genes are induced mostly in the epithelium at T1 samples (1 day post-IR). (C) Whole-mount view of feather epithelial branching after protein delivery into the feather follicles in vivo. Samples were collected 2 days post-treatment. In control experiments, BSA protein was used. Representative data from at least three experiments were shown. Bar = 100 µm.

An important consequence of IR exposure is activation of cytokine production. This is often associated with long-term effect of IR exposure such as inflammation and fibrosis [11]–[14]. We checked cytokine gene expression in the feather follicles after IR exposure (Fig. 4A and Fig. S4). Among the genes analyzed, IL-1beta, IFN-gama and TNF-alpha showed increased expression. TGF-beta1 is often implicated in other systems including the skin and lung [42], [43]. Here we found TGF-beta1 is actually not expressed in the feather follicle, while TGF-beta2/TGF-beta3 expression was not increased. We confirmed the increased expression of these genes by *in situ* hybridization (Fig. 4B).

To evaluate the possible contribution of these cytokines, we delivered these molecules into the feather follicles using an agarose bead method [36]. This protocol allows direct visualization of the impact of cytokine proteins on feather branching formation in vivo. A control bead coated with BSA produced no phenotype, as expected (Fig. 4C). In contrast, IL-1, IFN-gama and TNF-alpha coated beads disrupted the regular feather branching. These results suggest that the increased expression of cytokine genes could contribute to the disrupted feather branching.

### Stat1 is Involved in IR-induced Defects in Feather Formation

**Figure 5:**
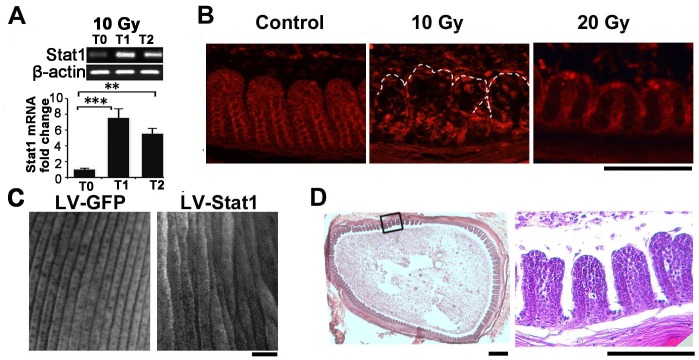
Involvement of Stat1 gene in IR-induced feather defects. (A) RT-PCR and densitometrical quantification showing increased Stat1 expression in the feather follicles. **, p<0.01; ***, p<0.001. (B) Stat1 antibody staining showing nuclear enrichment after IR exposure (T1 samples). (C) Whole-mount view of the feather epithelium after lentiviral-mediated Stat1 over-expression in the feather follicle. In control experiments, a lentivirus carrying GFP was used. (D) H&E analysis of Stat1 over-expressed feather follicle showing abnormal epithelial branching. Samples were collected 2 weeks after virus infection. Bar = 100 µm.

Cytokine molecules could induce complex responses in the cell signaling network. To evaluate the contribution of downstream signaling pathways, and more importantly, to enable rationally design methods to reduce or rescue the IR damage, we investigated the downstream events. An important route for cytokine signal transduction is the Jak/Stat1 pathway. We found IR exposure significantly increased Stat1 expression (Fig. 5A). Immunohistochemistry revealed increased staining, particularly nuclear enrichment, in the feather epithelium (Fig. 5B). These results promoted us to inquiry the role of this gene in feather branching formation.

To this end, we developed methods based on lentiviral delivery to over-express or knockdown gene expression in the feather follicle. This method allows wide-spread and long-term gene expression (Chu et al., submitted). Compared to a control virus carrying only GFP, Stat1 over-expression produced extensive branching abnormality (Fig. 5C). This abnormality can also be identified in H&E sections (Fig. 5D). Therefore, Stat1 gene could play an important role in mediating IR-induced defects in feather formation.

### AG-490, a Chemical Inhibitor of Stat1 Signaling, Rescues IR-induced Defects in Feather Formation

**Figure 6:**
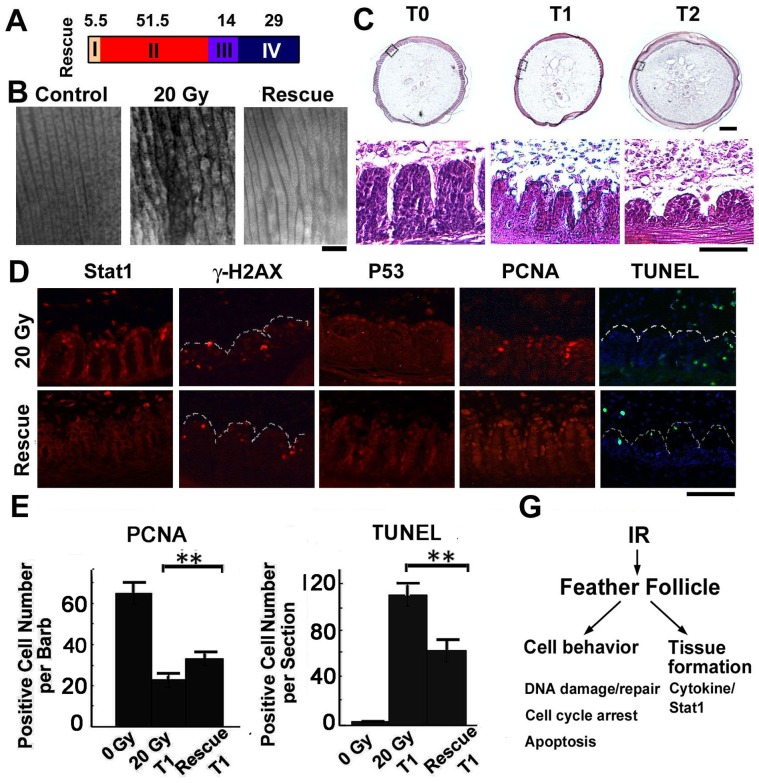
AG-490 partially rescues IR-induced defects in feather formation. (A) Statistics of feather morphology after 20 Gy IR exposure and AG-490 rescue. Compared to un-rescued samples shown in Figure 1, the feather morphology was significantly improved (n = 57). (B) Whole-mount view of feather branching and (C) H&E staining showing the improved feather morphology after AG-490 rescue. Epithelial branching was retained at both T1 and T2. (D) Molecular analysis of AG-490 rescue at T1. Notice the reduced nuclear Stat1, similar P53/gama-H2AX, increased PCNA, and reduced TUNEL staining. (E) Statistics of PCNA and (F) TUNEL staining results. **, p<0.01. (G) Summary of IR-responses in the feather follicle. A cytokine/Stat1 cascade disrupts the normal patterning event in the feather epithelium. Bar = 100 µm.

Supporting evidence for a role of Stat1 gene in IR pathology comes from rescue experiments. AG-490 is a small molecule inhibitor of Jak/Stat1 signaling. We tested whether this molecule could reduce or rescue IR-induced defects in feather formation. Indeed at a dose of 5 mg/kg, twice i.p. injection of AG-490 at T0 and T1 significantly improved the feather morphology. The most severe case, grade IV defect was reduced from 70% to 29%. Less severe case, grade II defect was increased from 18% to 51.5%. And 5.5% cases showed only grade I defect (Fig. 6A; n = 57). AG-490 by itself does not disrupt feather formation. The specificity of AG-490 treatment was examined in vivo in the feather follicles. As expected, AG-490 treatment reduced Stat1 expression both at the mRNA and the protein levels. However, the closely related molecules including Stat3 and Erk phosphorylation were not changed (Fig. S5).

The rescue effect of AG-490 was further analyzed by whole-mount prep. Compared to un-rescued samples, the feather branching was more regular although some defects still remained (Fig. 6B). H&E staining revealed some epithelial branching was retained at T1 and T2 (Fig. 6C), as compared to un-rescued control samples (Fig. 2B). Immunohistochemistry analysis revealed reduced expression, particularly loss of nuclear localization of Stat1 protein, suggesting reduced Stat1 signaling by AG-490 (Fig. 6D). Similar expression of gama-H2AX and P53 were observed, suggesting unchanged early responses. PCNA staining was increased by AG-490 rescue (Fig. 6E), while TUNEL was decreased (Fig. 6F). In summary, AG-490 treatment partially rescued the feather defects, helped maintain the regular epithelial branching, and retained more cells by increased cell proliferation and reduced cell apoptosis.

## Discussion

IR is routinely used in treating cancer patients. A wide range of cell and tissue behaviors are affected by this treatment. As shown here in the feather follicle, activation of P53, induction of DNA damage and repair, cell cycle arrest and apoptosis, cytokine production, and ablation of melanocyte stem cells, etc. all contribute to IR-induced tissue damage. To evaluate the specific contribution of these events is often difficult, partly because the biological systems have tremendous repair and re-adjustment capability that will sometimes cover-up the damage. For example, we find extensive PARP activation in response to 5 Gy or higher dose exposure. Previous reports suggested IR could induce extensive DNA damage and double-strand break, but mostly will be repaired within an hour [44]. We also noticed extensive patterning defects in feather branching after 10 Gy exposure. However, in the final feather morphology only 10% showed noticeable abnormality. This is probably due to adjustment in branching growth in later phases of feather development. On the other hand, IR-induced feather whitening seems to be irreversible, because the feathers in the next few cycles remain white. Previous work showed that IR-induced hair graying is due to P53-dependent depletion of melanocyte stem cells [45]. It seems the feather follicles were unable to repair the IR-damaged pigmentation system.

A clear dose-response to IR is revealed by this investigation. At 5 Gy, although P53 activation/gama-H2AX/PARP expression was induced, no cell cycle arrest or apoptosis happened. No reduction in cell number was noticed. This may due to a threshold mechanism, because P53 activation seems weaker and more transient at this dose. However, at 10 Gy still we noticed no reduction in cell number. Activation of P53/gama-H2AX/PARP all seemed strong and durable, yet cell cycle arrest and apoptosis was not seen. Reduction in cell number is only seen at an even higher dose (20 Gy) and is accompanied by cell cycle arrest and apoptosis. Therefore, a critical dose is required to achieve a desired damage level.

The patterning defect induced by IR in feather formation is intriguing. Such an effect has long been suspected but the exact mechanism remains elusive, as IR induced many defects in embryonic development. The progress zone model is among the proposed mechanisms. However, recent work suggested that IR-ablation of progenitor cells but not a patterning mechanism is responsible [28]. Here in the feather system, we provide evidence that a direct patterning effect is possible. Partitioning the epithelial cells into barb branches is the basic event for feather morphogenesis. Although the exact mechanism remains incompletely explored, BMP/Shh and Wnt signaling are involved [35]. Our recent work suggested FGF [37] and Notch signaling may also involve (unpublished data). Nonetheless, we showed that by beads coated protein perturbation, cytokines could disrupt the regular feather branching pattern. Furthermore, Stat1 over-expression or AG-490 inhibition could also promote or inhibit abnormalities in feather formation. These results strongly suggest that cytokine signal induced by IR could directly cause patterning defects in the feather follicle.

The feather system is a traditional model for developmental studies. The application of new experimental tools further fuels this system. A whole-mount open prep allows direct visualization of the branching events. Protein coated beads could directly perturb feather growth or branching formation in vivo and is easy to operate. More recently, novel methods based on lentiviral delivery to over-express or knockdown gene expression was developed (Chu et al., unpublished work), in addition to the more traditional RCAS-mediated gene delivery methods. These progresses provide additional power for the system. The exquisite branching morphology is ideal for a “recording” purpose. Any perturbation during development, either physiologically or experimentally, will be exhibited in the final morphology. This property makes it a wonderful system for pathological studies such as applied here in the IR settings. For instance, IR induces oxidative and nitrative stresses. iNOS [14], PI-3K/AKT [46] and MAPK [47] pathways are among the IR-induced responses. In the future, it will be interesting to test in the feather follicles the potential roles of these events in IR-induced tissue damage.

Through this investigation, we find AG-490, a small molecule inhibitor of Jak/Stat1 signaling, can rescue IR-induced tissue damage. Stat1 signaling is often involved in cytokine production, inflammation and immune regulation [48]. Here we find it could directly regulate epithelial patterning. The feather morphology is significantly improved by AG-490 rescue. More cells are retained in the feather follicle, with more cell proliferation and reduced cell death. Given that there are currently very few options to ameliorate IR-induced tissue damage [3], our discovery suggests AG-490 may worth further test in animal models and/or in clinic settings.

## Materials and Methods

### Experimental Animal

Three to six months old chickens were purchased from a local farm and housed in the Animal Facility Center in Fuzhou University. A 12-hour diurnal period was applied, and birds had free access to water and food. All operations and procedures were approved by the Animal Research Committee of Fuzhou University.

### IR Exposure of the Chicken and Rescue Experiments

The chickens were anesthetized with pentobarbital (50 mg/kg) before IR exposure in Department of Radiation Oncology, Union Hospital of Fujian Medical University. A Varian Clinac 23 ex machine (linear accelerator) was used to provide the 9 MeV electron beam. A lead cover was used to protect the rest of the body, with a 25 cm×25 cm window to expose the chicken wing. A homogeneous distribution of IR was achieved in this exposure window. The radiation was given at an intensity of 500 cGy/min, a dose-rate we routinely used to treat patients. It usually takes 1–4 min to finish the exposure. After exposure, the chickens were returned to the housing facility and cared. For rescue experiment, AG-490 (Beyotimes) was dissolved in DMSO, diluted in 8.0 ml sterile PBS, and i.p. injected twice at 5 mg/kg right before IR exposure and 24 hours later.

### Feather Growth Induction, Sample Collection and Photograph

To induce active growth, feathers were plucked in the wing contour region. After 2 weeks, the feather follicles entered growth phase. Feather follicles were collected before (control) or after IR exposure at designated times and processed for further analysis. For documentation of the gross morphology, feathers were collected after finishing the growth cycle and photographed using a HP scanner.

### Open Prep of the Feather Follicle

Open prep of the feather follicle was described previously [36]. Briefly, the feather follicle was cut open under a dissection microscope, and the mesenchymal pulp was removed. The remaining epithelial sheath was fixed by 4% PFA in PBS at 4°C overnight, counter stained by 1 ug/ml DAPI in PBS for 1 hour at room temperature, briefly washed in 3X PBS, and mounted for photograph under an inverted Nikon fluorescence microscope.

### Histology, Immunohistochemistry, and TUNEL Staining

H&E staining, immunostaining and in situ hybridizations were processed as described [36]. Briefly, feather follicles were fixed by 4% PFA in PBS at 4°C overnight, and processed for paraffin section. Eight um sections were collected for analysis. For antibody staining, we performed antigen retrieval using the 0.1 M citric acid buffer PH6.0 protocol and boiled the slides for 15 min. The following antibodies were used: P53, PARP, PCNA, Caspase-3, Stat1 (Santa Cruz), gama-H2AX (Abcam). For TUNEL staining, a commercial kit from Beyotimes was used and instructions followed. Briefly, paraffin sections were hydrolyzed and digested with 20 ug/ml proteinase K at 37°C for 15 min. After 3X PBS wash, TdT enzyme and FITC-dUTP reaction buffer was applied to the slide and incubated for 60 min at 37°C. After 3X additional wash with PBS, slides were counter stained with DAPI, mounted and photographed under a Leica fluorescence microscope. Quantification of the staining results was performed by counting positive cells per area by three independent investigators.

### RT-PCR Analysis

An average of four feather follicles were collected from the chicken at designated times, and total RNAs were extracted using the Trizol reagent (Shanghai Sangon). RNA quality was monitored by electrophoresis. PCR was performed using a pre-mix from CWBIO, Beijing. The conditions used were 5 min at 95°C, 29–38 cycles at 94°C for 30 sec, 60°C for 30 sec, 72°C for 30 sec, followed by 72°C for 7 min. Primers for each gene were individually verified for the correct size of the amplicon. Equal loading was monitored by endogenous β-Actin gene expression. Primer sequences available upon request.

### In Situ Hybridization

We cloned a segment of the respective chicken genes into pUCmT vector (Shanghai Sangon), and sequenced to confirm the correct gene and direction. Digoxingenin labeled RNA probe was prepared by in vitro transcription using a commercial kit from Roche. In situ hybridization was performed as described previously [36]. BM purple substrate (Roche) was used to develop color for the Alkaline phosphatase coupled anti-Dig antibody (Roche).

### Implantation of Protein Coated Beads

DEAE sepharose beads (BBI) was washed in PBS twice, and mixed with the desired protein at a final concentration of 1 ng/ul. Beads were absorbed at room temperature for 1 hour, and implanted into the developing feather follicle in vivo [36]. The chickens were anesthetized with pentobarbital (50 mg/kg) during the operation.

### Lentiviral-mediated Gene Delivery in the Feather Follicle

Lentivirus were produced and harvested in HEK 293T cells using the standard protocol. Full length mouse Stat1 gene (IMAGE) was cloned into pLVX-ZxGreen (a gift from Dr Jun Xu, Tongji University, Shanghai, China) and produced lentivirus. Virus transfection of regenerative feathers and sample processing were performed as described [35]. Briefly, plucked feather follicles were washed with PBS, and virus supernatant injected immediately. Total injection volume is about 80–120 ul per each follicle. To reduce variation in the experiment and avoid bleeding after plucking, only flight feathers in their resting phase were used.

### Statistical Analysis

Semi-quantitative PCR and Western blot analysis results were densitometrically quantified using ImageJ 1.44 P after at least three independent experiments. Data were expressed as mean ± standard deviation. The statistical difference between two groups was determined by two-tailed t-test. The statistical significance (p-value) was calculated and described. A p value of less than 0.05 was considered to be statistically significant (*), p less than 0.01 was marked by (**), and 0.001 (***).

## Supporting Information

Figure S1 **Regeneration of the feather follicle after 20 Gy IR exposure.** (A) All feather follicles can regenerate in the next growth cycle, but mostly showed a loss of pigmentation. (B) Feathers showing “albinism” as compared to control feathers in the unexposed area. Bar = 1 cm.
(PDF)

Figure S2 **5 Gy IR exposure does not induce abnormality in feather formation.** Compared to a control sample at T0, 5 Gy IR treated feather follicles remained normal at T1 and T2. Five feather follicles were examined by H&E staining in each case and representative samples are shown. T0, untreated control; T1, 1 day post-IR; T2, 2 days post-IR. Bar = 100 µm.
(PDF)

Figure S3 **Molecular analysis in the feather follicles after 5 Gy IR exposure.** (A) Immunohistrochemistry. Notice the activation of P53, gama-H2AX and PARP at T1, but attenuated at T2. PCNA and Caspase-3 staining were unchanged. Bar = 100 µm. (B) RT-PCR analysis of gene expression in the feather follicles. Each experiment was repeated at least three times, and the results were densitometrically quantified and statistically analyzed. *, p<0.05; **, p<0.01. T0, untreated control; T1, 1 day post-IR; T2, 2 days post-IR.
(PDF)

Figure S4 **Quantification of molecular expression in the feather follicles.** RT-PCR analysis of gene expression in the feather follicles were densitometrically quantified (for P53/P21/Cyclin D1/Fas gene, gels were shown in Fig. 3D) and statistically analyzed. *, p<0.05; **, p<0.01; ***, p<0.001. T0, untreated control; T1, 1 day post-IR; T2, 2 days post-IR.
(PDF)

Figure S5 **Specificity of AG-490 treatment in the feather follicles.** (A–B) Western blot analysis; (C–D) RT-PCR analysis of the feather follicles after 20 Gy IR exposure, with or without AG-490 rescue (5 mg/kg i.p. twice injection at T0 and T1). T0 samples were used as control (no IR exposure). Results were densitometrically quantified and statistically analyzed. *, p<0.05; **, p<0.01. T1, 1 day post-IR; T2, 2 days post-IR. Note the chicken pErk shows only 1 band, as previously reported (Trimarchi T et al. J Neurochem. 108∶246–259, 2009; Duchene S et al. Domest. Anim. Endocrinol. 34∶63–73, 2008).
(PDF)
