# Supplementary material for: Dissecting the Molecular Mechanism of Ionizing Radiation-Induced Tissue Damage in the Feather Follicle
Source: PLoS One. 2014 Feb 20;9(2):e89234. doi: 10.1371/journal.pone.0089234 (PMC3930710; doi:10.1371/journal.pone.0089234)
Supplement: Figure S1 — Regeneration of the feather follicle after 20 Gy IR exposure. (A) All feather follicles can regenerate in the next growth cycle, but mostly showed a loss of pigmentation. (B) Feathers showing “albinism” as compared to control feathers in the unexposed area. Bar = 1 cm. (PDF) [file pone.0089234.s001.pdf]

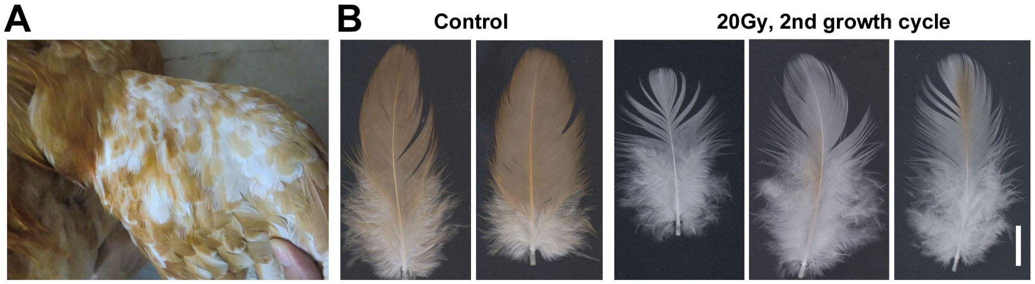

Figure S1. Regeneration of the feather follicle after 20Gy IR exposure.

(A) All feather follicles can regenerate in the next growth cycle, but mostly showed a loss of pigmentation. (B) Feathers showing “albinism” as compared to control feathers in the unexposed area. Bar=1cm.
