# Supplementary material for: Dissecting the Molecular Mechanism of Ionizing Radiation-Induced Tissue Damage in the Feather Follicle
Source: PLoS One. 2014 Feb 20;9(2):e89234. doi: 10.1371/journal.pone.0089234 (PMC3930710; doi:10.1371/journal.pone.0089234)
Supplement: Figure S2 — 5 Gy IR exposure does not induce abnormality in feather formation. Compared to a control sample at T0, 5 Gy IR treated feather follicles remained normal at T1 and T2. Five feather follicles were examined by H&E staining in each case and representative samples are shown. T0, untreated control; T1, 1 day post-IR; T2, 2 days post-IR. Bar = 100 µm. (PDF) [file pone.0089234.s002.pdf]

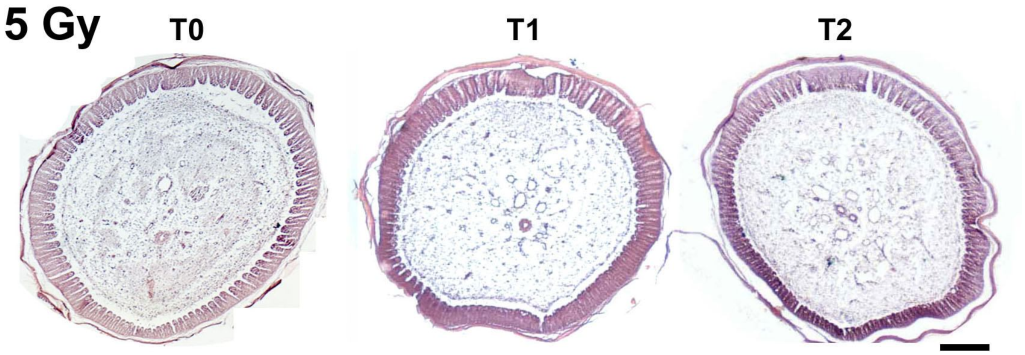

Figure S2. 5Gy IR exposure does not induce abnormality in feather formation.

Compared to a control sample at T0, 5Gy IR treated feather follicles remained normal at T1 and T2. Five feather follicles were examined by H&E staining in each case and representative samples are shown. T0, untreated control; T1, 1 day post-IR; T2, 2 days post-IR. Bar=100μm.
