# Supplementary material for: Dissecting the Molecular Mechanism of Ionizing Radiation-Induced Tissue Damage in the Feather Follicle
Source: PLoS One. 2014 Feb 20;9(2):e89234. doi: 10.1371/journal.pone.0089234 (PMC3930710; doi:10.1371/journal.pone.0089234)
Supplement: Figure S3 — Molecular analysis in the feather follicles after 5 Gy IR exposure. (A) Immunohistrochemistry. Notice the activation of P53, gama-H2AX and PARP at T1, but attenuated at T2. PCNA and Caspase-3 staining were unchanged. Bar = 100 µm. (B) RT-PCR analysis of gene expression in the feather follicles. Each experiment was repeated at least three times, and the results were densitometrically quantified and statistically analyzed. *, p<0.05; **, p<0.01. T0, untreated control; T1, 1 day post-IR; T2, 2 days post-IR. (PDF) [file pone.0089234.s003.pdf]

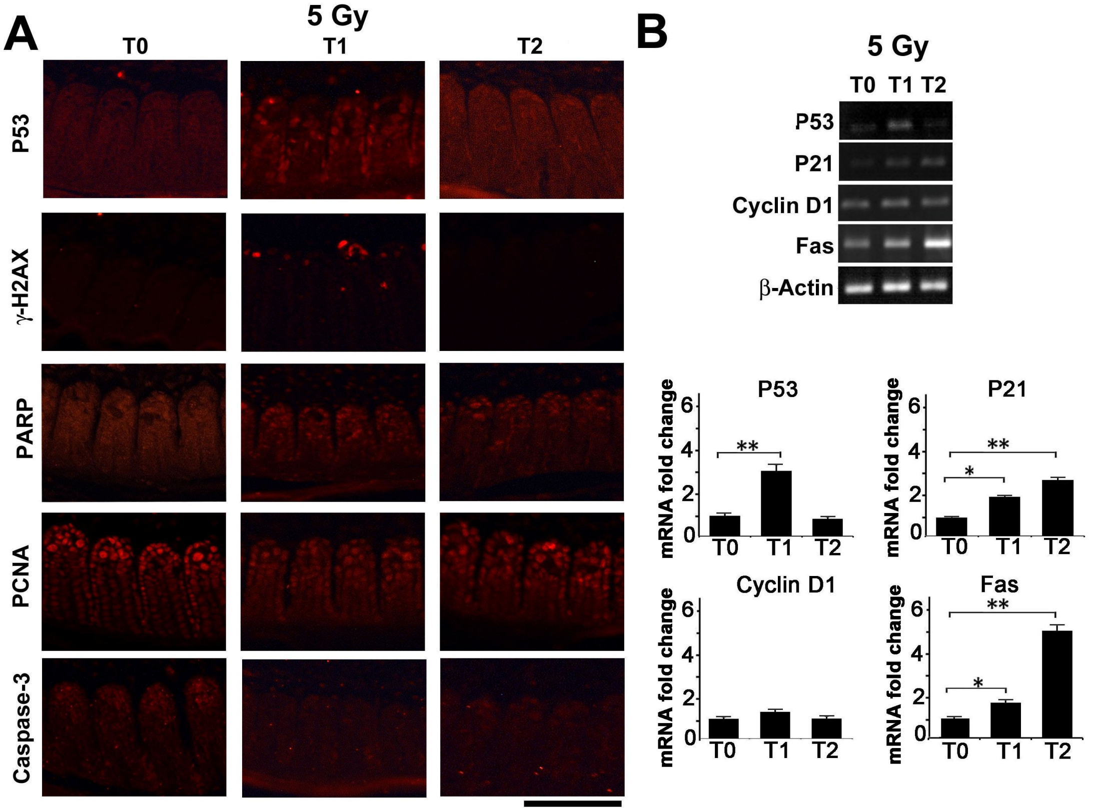

Figure S3. Molecular analysis in the feather follicles after 5Gy IR exposure.

(A) Immunohistrochemistry. Notice the activation of P53, gama-H2AX and PARP at T1, but attenuated at T2. PCNA and Caspase-3 staining were unchanged. Bar=100μm. (B) RT-PCR analysis of gene expression in the feather follicles. Each experiment was repeated at least three times, and the results were densitometrically quantified and statistically analyzed. *, $p<0.05$; **, $p<0.01$. T0, untreated control; T1, 1 day post-IR; T2, 2 days post-IR.
