# Supplementary material for: Dissecting the Molecular Mechanism of Ionizing Radiation-Induced Tissue Damage in the Feather Follicle
Source: PLoS One. 2014 Feb 20;9(2):e89234. doi: 10.1371/journal.pone.0089234 (PMC3930710; doi:10.1371/journal.pone.0089234)
Supplement: Figure S4 — Quantification of molecular expression in the feather follicles. RT-PCR analysis of gene expression in the feather follicles were densitometrically quantified (for P53/P21/Cyclin D1/Fas gene, gels were shown in Fig. 3D) and statistically analyzed. *, p<0.05; **, p<0.01; ***, p<0.001. T0, untreated control; T1, 1 day post-IR; T2, 2 days post-IR. (PDF) [file pone.0089234.s004.pdf]

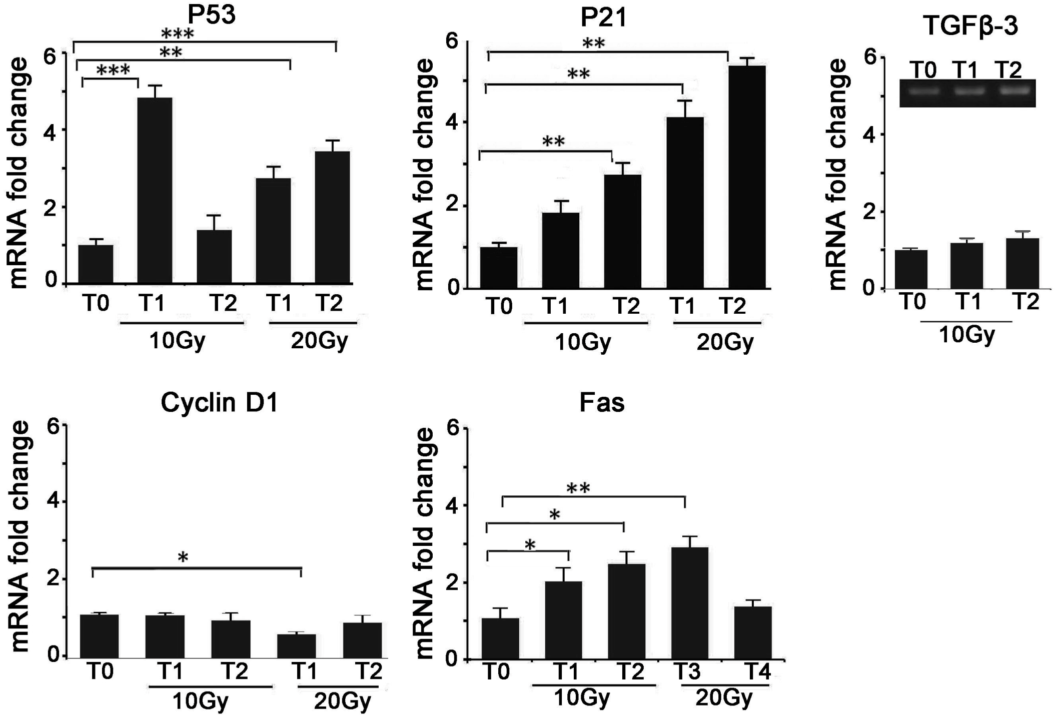

Figure S4. Quantification of molecular expression in the feather follicles.

RT-PCR analysis of gene expression in the feather follicles were densitometrically quantified (for P53/P21/Cyclin D1/Fas gene, gels were shown in Fig. 3D) and statistically analyzed. *, $p<0.05$; **, $p<0.01$; ***, $p<0.001$. T0, untreated control; T1, 1 day post-IR; T2, 2 days post-IR.
