# Supplementary material for: Dissecting the Molecular Mechanism of Ionizing Radiation-Induced Tissue Damage in the Feather Follicle
Source: PLoS One. 2014 Feb 20;9(2):e89234. doi: 10.1371/journal.pone.0089234 (PMC3930710; doi:10.1371/journal.pone.0089234)
Supplement: Figure S5 — Specificity of AG-490 treatment in the feather follicles. (A–B) Western blot analysis; (C–D) RT-PCR analysis of the feather follicles after 20 Gy IR exposure, with or without AG-490 rescue (5 mg/kg i.p. twice injection at T0 and T1). T0 samples were used as control (no IR exposure). Results were densitometrically quantified and statistically analyzed. *, p<0.05; **, p<0.01. T1, 1 day post-IR; T2, 2 days post-IR. Note the chicken pErk shows only 1 band, as previously reported (Trimarchi T et al. J Neurochem. 108∶246–259, 2009; Duchene S et al. Domest. Anim. Endocrinol. 34∶63–73, 2008). (PDF) [file pone.0089234.s005.pdf]

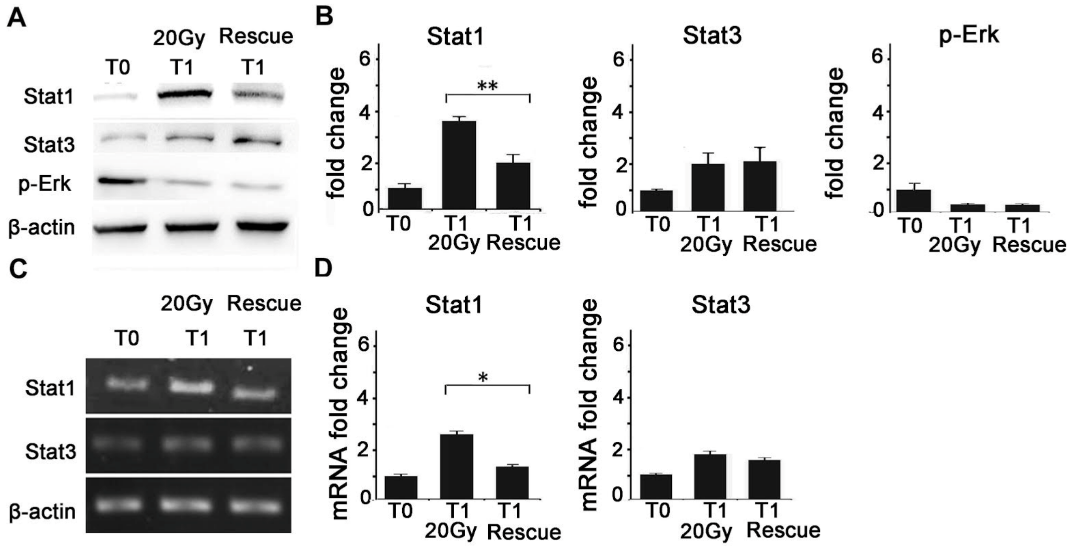

Figure S5. Specificity of AG-490 treatment in the feather follicles.

(A-B) Western blot analysis; (C-D) RT-PCR analysis of the feather follicles after 20Gy IR exposure, with or without AG-490 rescue (5mg/kg i.p. twice injection at T0 and T1). T0 samples were used as control (no IR exposure). Results were densitometrically quantified and statistically analyzed. *, $p<0.05$; **, $p<0.01$. T1, 1 day post-IR; T2, 2 days post-IR. Note the chicken pErk shows only 1 band, as previously reported (Trimarchi T et al. J Neurochem. 108: 246–259, 2009; Duchene S et al. Domest. Anim. Endocrinol. 34: 63–73, 2008).
